# Ventilatory efficiency testing as prognostic value in patients with pulmonary hypertension

**DOI:** 10.1186/1471-2466-12-23

**Published:** 2012-06-07

**Authors:** Martin Schwaiblmair, Christian Faul, Wolfgang von Scheidt, Thomas M Berghaus

**Affiliations:** 1 Department of Internal Medicine I, Klinikum Augsburg, Ludwig Maximilians University of Munich, Munich, Germany

## Abstract

**Background:**

Increased ventilatory response has been shown to have a high prognostic value in patients with chronic heart failure. Our aim was therefore to determine the ventilatory efficiency in pulmonary arterial hypertension and chronic thromboembolic pulmonary hypertension by cardiopulmonary exercise testing (CPET) identifying PH-patients with increased risk for death within 24 months after evaluation.

**Methods:**

116 patients (age: 64 ± 1 years) with a mean pulmonary arterial pressure of 35 ± 1 mmHg underwent CPET and right heart catheterization. During a follow-up of 24 months, we compared the initial characteristics of survivors (n = 87) with nonsurvivors (n = 29).

**Results:**

Significant differences (p ≤ 0.005) between survivors and nonsurvivors existed in ventilatory equivalents for oxygen (42.1 ± 2.1 versus 56.9 ± 2.6) and for carbon dioxide (Ve/VCO2) (47.5 ± 2.2 versus 64.4 ± 2.3). Patients with peak oxygen uptake ≤ 10.4 ml/min/kg had a 1.5-fold, Ve/VCO2 ≥ 55 a 7.8-fold, alveolar-arterial oxygen difference ≥ 55 mmHg a 2.9-fold, and with Ve/VCO2 slope ≥ 60 a 5.8-fold increased risk of mortality in the next 24 months.

**Conclusions:**

Our results demonstrate that abnormalities in exercise ventilation powerfully predict outcomes in PH. Consideration should be given to add clinical guidelines to reflect the prognostic importance of ventilatory efficiency parameters in addition to peak VO2.

## Background

Most patients with pulmonary hypertension (PH) have exercise limitation attributing to an impaired vasodilator response of the pulmonary arteries during exercise. Cardiopulmonary exercise testing (CPET) describes the underlying physiologic abnormalities associated with the underperfusion of the pulmonary vascular bed seen in PH [1-6]. Additionally, CPET allows reproducible assessment of functional capacity and treatment efficacy in patients with PH [2,5,7-10].

Peak oxygen uptake (VO2) is the most frequently analyzed CPET parameter and has consistently demonstrated prognostic significance. Other ventilatory expired gas parameters obtained during exercise testing have recently demonstrated prognostic value. The relationship between minute ventilation and carbon dioxide production (Ve/VCO2 slope) is one of such CPET parameter that appears to have clinical value. It can be derived from submaximal exercise testing and is independent of patient motivation. The Ve/VCO2 slope is inversely related to cardiac output at peak exercise [11] and is at least partly explained by a decrease in pulmonary perfusion [12,13]. Ventilatory efficiency was found to be reliable predictor of prognosis in patients with chronic heart failure [14-22].

Since inefficiency of ventilation results from decreased perfusion of the ventilated lung, for example by an increased physiological dead space ventilation, a high ventilatory response relative to metabolic demand would be expected [23]. If a simple non-invasive measurement, such as the VE/VCO2 ratio, could be shown to be useful in evaluating pulmonary vascular disease, it might serve to supplement other methods currently used to monitor clinical course and treatment. At now, few studies have investigated the relationship between exercise capacity and VE/VCO2 slope in patients with PH [24,25].

Despite recent advances in pharmacological treatment of patients with PH, mortality rates, especially in patients with severe pulmonary artery hypertension (PAH), remain high. Reliable risk stratification is a continuing challenge and the identification of patients at highest risk for early death from right heart failure is of special importance. Functional capacity, as defined by peak VO2 during CPET is established as a powerful predictor of mortality in PAH [26,27] and a peak VO2 below 10.4 mL/kg/min is now considered a key criterion for early mortality [2]. To date however, differences in the prognostic value of peak VO2 and the Ve/VCO2 slope have not been widely emphasized.

In this study, we determine the predictive value of ventilatory efficiency parameters for estimating the 2-years survival of patients with pulmonary arterial hypertension (PAH) and inoperable chronic thromboembolic pulmonary hypertension (CTEPH) and compare these parameters with survival predicted by peak VO2.

## Methods

### Subjects

This study included consecutive patients who were referred to our PH centre from January 2005 to January 2008 to confirm and to classify the PH, as defined by the Proceedings of the 4^th^ World Symposium on Pulmonary Hypertension [26]. All patients underwent right heart catheterization (RHC) to establish the diagnosis, pulmonary function testing, six-minute walking test and a progressively increasing, symptom-limited CPET with gas exchange measurements. All patients did not receive any specific pulmonary artery medication at the beginning of the study. Patients with severe concomitant extracardiac diseases limiting exercise performance were excluded.

All patients were followed at the PH centre, Klinikum Augsburg, University of Munich. The outcome data were collected by patient`s visit every 3 months. The minimum follow-up period was 24 months.

All procedures adhered to the commonly accepted ethical guidelines, the protocol was initially reviewed and approved by an Ethics Committee and written informed consent was obtained from every patient.

### Lung function tests

Pulmonary function tests included spirometry, body plethysmography, and measurement of diffusing capacity employing the single breath method (Master Screen Body and MS-PFT, Jaeger, Cardinal Health, USA). Each parameter was calculated as percent of predicted [28]. The following parameters were determined: forced vital capacity (FVC), total lung capacity (TLC), forced expiratory volume in one second (FEV1) and diffusing capacity for carbon monoxide (TLco). Blood gas analysis (ABL 725, Radiometer, Copenhagen, Denmark) was performed in arterialized capillary blood from the ear lobe without supplemental oxygen [29].

### Six-minute walk test

Measurement of the distance walked in 6 min was performed in all patients according to the standards of the American Thoracic Society [30]. The patients were instructed to walk back and forth at their own pace in a 30-m corridor in order to cover as much ground as possible in the allotted time. A research assistant supervised the test, telling the patient the remaining exercise time every 2 min. The patients were allowed to stop and take at rest during the test, but were instructed to resume walking as soon as possible.

### Cardiopulmonary exercise testing

CPET was performed using standardized protocol [31]; work rate was continuously increased by 5 – 15 W/min to a maximum tolerated level on an electromagnetically braked cycle ergometer (ViaSprint 150 p, Ergoline, Germany). Patients were encouraged to exercise until symptoms were intolerable.

Blood gas analysis was analyzed at rest and during maximal exercise. Heart rate was monitored continuously, and non-invasive blood pressure was taken every 2 min. The maximum work rate was recorded. Oxygen uptake (VO2), minute ventilation (Ve), and carbon dioxide output (VCO2) were measured breath by breath wearing an adult facemask (Vmax spectra 229 D, Sensor Medics, CA, USA). Peak VO2, oxygen pulse (O2 pulse) and alveolar-arterial oxygen difference (AaDO2) were calculated as described by Wasserman et al. [31]. The anaerobic threshold (AT) was chosen as the VO2 at which the Ve/VO2 increased while the Ve/VCO2 decreased or remained constant. Peak VO2 was defined as the value of averaged data during the final 15 seconds of exercise. The Ve/VCO2 slope was determined as the linear regression slope of Ve and VCO2 from the start of exercise until the RC point (the time at which ventilation is stimulated by CO2 output and end-tidal CO2 tension begins to decrease) [31].

### Right heart catheterization

All patients underwent RHC. Patients received no medications on the morning of the procedure, resulting in a discontinuation of treatment with medication of about 12 h. A True Size Thermodilution Catheter (“S” Tip Catheter, Edwards Lifesciences, Irvine, CA, USA) was inserted via the right femoral vein. Hemodynamic measurements were performed in supine position and included heart rate, pressure in wedge-position (PCWP), pulmonary arterial pressure (PAP) and right atrium pressure (RA). Oxygen saturation (SO2) was measured in mixed venous blood samples (ABL 725, Radiometer, Copenhagen, Denmark). Cardiac index (CI) was obtained using the thermodilution method (Com-2, Cardiac Output Computer, Edwards Lifesciences, Irvine, CA, USA). CI and pulmonary vascular resistance (PVR) were calculated using standard formulas [PVR = (mPAP – PCWP)/Q].

### Statistical analysis

Continuous date were presented as the mean ± standard error of mean (SEM); categorical data were presented as percentages of patients in each category. A statistical software package (SPSS, version 12.0 for Windows; SPSS; Chicago, IL) was used for analysis. All results were tested for two-sided significance. Patients were divided in two different groups regarding the outcome after 24 months. The measured variables were compared in the group of survivors versus the group of nonsurvivors using the Student *t*-test for unpaired probes.

Simple linear regression analysis was used to examine ve versus VCO2 slope. Test analysis was performed to calculate sensitivity, specificity for the different threshold values.

Furthermore, we used the Wald test to test for the significance of odds ratio. The ability of CPET parameters to identify the risk of mortality was assessed by comparison of receiver-operating characteristic curves (AUC = area under curve). We create survival curves using the method of Kaplan and Meier and compare the survival curves using the log-rank test.

In general, p-values < 0.05 were considered to be statistically significant.

## Results

### Clinical characteristics

The study included 116 patients; there were 73 women and 43 men with a mean age of 63.7 ± 1.7 years and a body mass index of 26.7 ± 0.4 kg/m². According to the ESC/ERS guidelines [27], we diagnosed 85 patients as PAH and 31 as inoperable CTEPH [Table 1. At the initial visit, all patients fulfilled the criterion to begin a specific drug therapy. During follow-up, the specific drug therapy consisted calcium channel blockers in 7%, inhaled prostanoids in 13%, endothelin receptor antagonists in 72% and 56% patients were treated with phosphodiesterase type-5 inhibitors. None of the patients was lost to follow-up. During the follow-up period of 24 months, 29 patients died (n = 21 in the PAH-group and n = 8 in the CTEPH-group). Causes of death were progressive right-heart failure (n = 12), respiratory failure (n = 10), pulmonary hemorrhage (n = 2), arrhythmia (n = 2) and others (n = 3).

**Table 1:** Characteristics, haemodynamics and Cardiopulmonary exercise parameters at baseline of the overall group (n = 116) and between patients with PAH (n = 85) and those with CTEPH (n = 31)

	**Overall**	**PAH**	**CTEPH**	**p**
	**(n = 116)**	**(n = 85)**	**(n = 31)**	
Age (years)	61.5 ± 1.5	59.9 ± 1.9	66.4 ± 2.0	p = 0.064
Sex (% female)	66	70	55	ns
BMI (kg/m2)	25.8 ± 0.5	25.7 ± 0.6	26.2 ± 0.7	ns
PAP, mmHg	39.9 ± 2.5	40.5 ± 1.7	38.3 ± 3.1	ns
CI, L*min-1*m-2	2.48 ± 0.07	2.58 ± 0.08	2.22 ± 0.11	p = 0.021
PVR, dynes * s * cm-5	648.2 ± 40.2	647.9 ± 45.1	648.9 ± 87.5 ns	
RAP, mmHg	7.28 ± 0.49	7.01 ± 0.56	8.07 ± 1.00	ns
W, watts	56.2 ± 3.1	55.2 ± 3.7	59.1 ± 5.5	ns
VO2, ml/min/kg	14.4 ± 0.7	14.5 ± 0.8	14.1 ± 1.2	ns
AT, ml/min/kg	9.53 ± 0.40	9.56 ± 0.48	9.45 ± 0.72	ns
O2 pulse, ml/min/beat	8.98 ± 0.36	8.71 ± 0.40	9.72 ± 0.81	ns
Ve/VO2 - AT	41.1 ± 1.1	40.9 ± 1.2	41.8 ± 2.3	ns
Ve/VCO2 - AT	47.2 ± 1.1	47.0 ± 1.2	47.8 ± 2.5	ns
AaDO2 (peak exercise)	mmHg 49.3 ± 1.6	51.6 ± 1.9	43.2 ± 2.9	p = 0.019
a-et CO2, mmHg	5.7 ± 0.5	5.2 ± 0.5	7.0 ± 1.0	ns
Ve/VCO2 slope	46.9 ± 2.0	46.6 ± 2.3	47.9 ± 3.9	ns

*W* = work capacity.

*VO2* = peak oxygen uptake.

*AT* = anaerobic threshold.

*O2 pulse* = oxygen pulse.

*Ve/VO2* = oxygen equivalent at anaerobic threshold.

*Ve/VCO2* = carbon dioxide equivalent at anaerobic threshold.

*AaDO2* = alveolar-arterial oxygen difference at peak exercise.

*a-et CO2* = arterial-endexpiratory carbon dioxide difference at peak pexercise.

*Ve – VCO2* slope = slope of minute ventilation versus carbon dioxide output.

### Lung function test

Lung function tests showed no relevant restrictive lung disease with FVC of 80.0 ± 1.5% and TLC of 91.0 ± 1.4%. The ratio of FEV1/FVC x 100 showed no signs of relevant obstructive lung disease in all patients (70.3 ± 0.9%). The diffusing capacity was slightly reduced, to 71.4 ± 2.1 predicted. Blood gas analysis showed a mild hypoxemia (pO2 of 63.6 ± 1.0 mm Hg) and hypocapnia (pCO2 of 33.8 ± 0.4 mm Hg).

### Hemodynamics

At initial visit and at rest, PAP was elevated (41.4 ± 2.5 mm Hg), consecutively with a PVR of 624 ± 70 dynes * s *cm-5, and with a normal PCWP of 9.6 ± 0.4 mmHg. The RAP was elevated with 7.6 ± 1.1 mm Hg.

### Cardiopulmonary exercise test and six minute walk distance

Most patients showed a reduction in work capacity of 58.8 ± 26.4% with a diminished aerobic capacity of 62.2 ± 2.1% (13.6 ± 1.3 ml/min/kg), a reduced O2 pulse of 9.3 ± 0.8 ml/min/beat and elevated ventilatory equivalents for oxygen (45.9 ± 2.2) and for carbon dioxide (51.8 ± 2.2) at the AT (9.0 ± 0.9 ml/min/kg). Furthermore, we observed elevated AaDO2 (56.5 ± 3.2 mmHg) during peak exercise with an elevated a-etCO2 - difference of 7.7 ± 0.9 mmHg. Ve/VCO2 slope amounted to 51.7 ± 4.3 with a PetCO2 of 28.0 ± 0.7 mmHg at peak exercise. The AT could not be determined in 11 patients because of mitigating oscillatory gas exchange patterns or premature end of exercise.

On average, we objectified a six minute walk distance of 314.5 ± 12.3 m or 62.4 ± 2.2% respectively.

### Evaluation of survivors and nonsurvivors in respect of CPET parameters in patients with pulmonary hypertension

In our study group, no significant differences in any of hemodynamic parameters, aerobic capacity, anaerobic threshold and oxygen pulse at baseline existed between survivors and nonsurvivors. On the other hand, significant differences could be demonstrated in the following parameters at baseline: power capacity (67.7 versus 35.7 watts, p = 0.023), Ve/VO2 (42.1 versus 56.9, p = 0.001), Ve/VCO2 (47.5 versus 64.4, p < 0.001), AaDO2 (52.8 versus 67.5 mmHg, p = 0.009) and Ve/VCO2 slope (47.0 versus 65.4, p = 0.014) [Table 2].

**Table 2:** Hemodynamics and Cardiopulmonary exercise characteristics at baseline between survivors and nonsurvivors in patients with selective drug therapy (survival after 24 months)

	**Survivors**	**Nonsurvivors**	**p**
	**(n = 87)**	**(n = 29)**	
PAP, mmHg	42.0 ± 2.5	39.6 ± 2.6	ns
CI, L*min-1*m-2	2.30 ± 0.12	2.31 ± 0.21	ns
PVR, dynes * s * cm-5	633.8 ± 68.2	594.1 ± 76.6	ns
RAP, mmHg	7.22 ± 0.79	8.55 ± 1.88	ns
W, watts	67.7 ± 8.0	35.7 ± 1.5	0.023
VO2, ml/min/kg	14.6 ± 1.4	10.9 ± 1.0	ns
AT, ml/min/kg	9.65 ± 0.87	7.09 ± 1.07	ns
O2 pulse, ml/min/beat	9.80 ± 0.81	7.79 ± 0.64	ns
Ve/VO2 - AT	42.1 ± 2.1	56.9 ± 2.6	0.001
Ve/VCO2 - AT	47.5 ± 2.2	64.4 ± 2.3	< 0.001
AaDO2 – peak exercise, mmHg	52.8 ± 2.8	67.5 ± 4.2	0.009
a-et CO2, mmHg	6.8 ± 0.8	10.5 ± 1.1	0.014
Ve/VCO2 slope	47.0 ± 3.2	65.4 ± 7.5	0.014

*PAP* = mean pulmonary artery pressure.

*CI* = cardiac index.

*PVR* = pulmonary vascular resistance.

*RAP* = mean right atrial pressure.

*W* = work capacity.

*VO2* = peak oxygen uptake.

*AT* = anaerobic threshold.

*O2 pulse* = oxygen pulse.

*Ve/VO2* = oxygen equivalent at anaerobic threshold.

*Ve/VCO2* = carbon dioxide equivalent at anaerobic threshold.

*AaDO2* = alveolar-arterial oxygen difference at peak exercise.

*a-et CO2* = arterial-endexpiratory carbon dioxide difference at peak pexercise.

*Ve – VCO2 slope* = slope of minute ventilation versus carbon dioxide output.

### Cardiopulmonary exercise parameters predictors of two-years mortality

**Figure 1:**
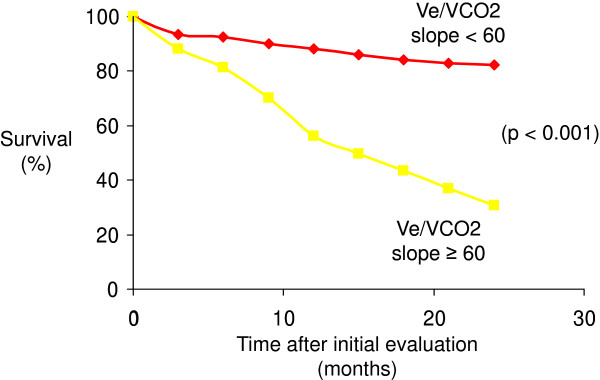
Kaplan-Meier plot relating survival to Ve/VCO2 slope.

**Figure 2:**
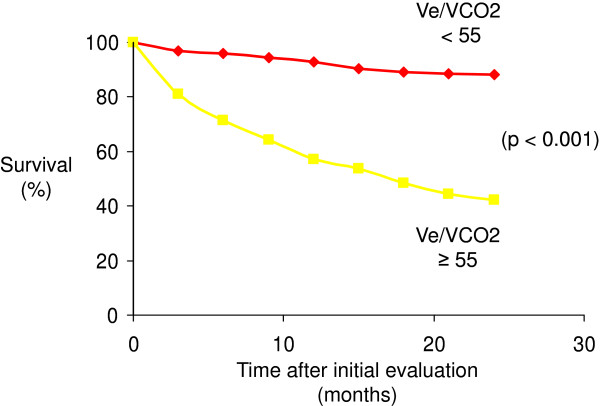
Kaplan-Meier plot relating survival to Ve/VCO2 at anaerobic threshold.

According to the study results from Wensel at al. [2], we firstly investigated the aerobic capacity with a cut-off value of 10.4 ml/min/kg. In our study population, VO2 (AUC = 0.252) could not predict the two-years mortality with a sensitivity of 27% and specificity of 69%. The best predictive value in our group was the Ve/VCO2 (with a cut-off value of 55, AUC = 0,769) with a relative risk of two-years mortality of 7.83 and a sensitivity of 80% and a specificity of 79% [Figure 1. Furthermore, Ve/VCO2 slope (AUC = 0.798) with a relative risk of 5.75 showed significant predictive mortality values [Figure 2. AaDO2 (AUC = 0.758) and a-etCO2 (AUC = 0.804) values at peak exercise was not able to predict the mortality because of a decreased specificity of 62%/69% [Table 3.

**Table 3:** Cardiopulmonary exercise parameters predictors of two-years mortality rate in patients in patients with selective drug therapy, ie, in patients with pulmonary artery hypertension and in patients with chronic thromboembolic pulmonary hypertension (n = 116)

	**Sensitivity**	**Specificity**	**odds ratio**	**significance**
			**[95% confidence interval]**	
VO2 (< 10.4 ml/min/kg)	27%	69%	1.88 [1,58; 2,18]	p = 0.03
Ve/VCO2 (≥ 55)	80%	79%	14.66 [12,70; 16,62]	p = 0.004
AaDO2 (≥ 55 mmHg)	69%	62%	3.67 [3,11; 4,23]	p = 0.09
Ve/VCO2 slope (≥ 60)	70%	81%	9.92 [6,68; 13,17]	p = 0.03
a-etCO2 (≥ 8 mmHg)	62%	69%	3.63 [2,73; 4,53]	p = 0.02

## Discussion

To our knowledge, this is one of the first studies of CPET data in patients with PAH/CTEPH to compare ventilatory efficiency parameters as a predictor of 24-months mortality. In our study, ventilatory equivalent for carbon dioxide at anaerobic threshold with a cutoff value of 55 identifies patients with an over seven fold increased risk of death within 24 months after initial evaluation. The Ve/VCO2 slope with a cutoff value of 60 had an over five fold increased risk of death within the same time interval. In our study group the parameters of ventilatory efficiency significantly better predicted the clinical outcome compared to peak oxygen uptake with a cut-off value of 10.4 ml/min/kg. Taken together, these results support the concept that for example the VE/VCO2 slope or the ventilatory equivalents may be prognostically superior to peak VO2.

Symptoms in PAH-patients develop during exercise because recruitment of pulmonary vascular bed needed for exercise is impaired. Three pathophysiologies could be easily identified: [1] failure to perfuse the ventilated lung, thereby increasing the physiologic dead space and ventilatory requirement [5,24,32]; [2] failure to increase cardiac output (oxygen transport) appropriately in response to exercise, causing a low work rate lactic acidosis (increased CO2 production relative to O2 consumption), thereby increasing acid ventilatory drive [11,33]; and [3] exercise-induced hypoxemia in most PH patients, thereby increasing the hypoxic ventilatory drive. Ventilatory inefficiency could be measured as an increase in ventilatory equivalents for oxygen and carbon dioxide. Reindl et al. [11] showed that ventilatory efficiency is influenced by cardiac output and by pulmonary vasoconstriction. Alveolar hypoperfusion, caused by impaired or defective vasodilation, probably represents the link to the impairment of ventilatory efficiency.

A significant finding in our study was the confirmation that Ve/VCO2 slope is a strong, independent predictor of death in patients suffering from PAH/CTEPH. This observation is in agreement with other studies from patients with chronic heart failure which have examined the prognostic significance of the Ve/VCO2 slope [15,19,34,35]. Patients with chronic heart failure ventilate more during exercise than controls, resulting in an increase in the Ve/VCO2 slope [36]. The increased Ve/VCO2 slope could be a result of different mechanism. Ventilation perfusion mismatch [12,37], impaired diffusion of metabolic gases [38], respiratory muscle weakness [39], and heightened sensitivity of peripheral receptors [40,41] have all been postulated as possible causes. Additionally, one of the proposed mechanisms for an increase in Ve/VCO2 slope in chronic heart failure patients is an abnormal pulmonary perfusion [42]. Furthermore, Ukkonen et al. [43] showed that the right ventricular oxidative metabolism correlates with Ve/VCO2 slope in chronic heart failure patients. This supports the hypothesis that pulmonary vascular resistance is a main determinant of the Ve/VCO2 slope [43]. Because of the increased pulmonary vascular resistance in PH, pulmonary blood flow (cardiac output) fails to increase normally during exercise. The blunted cardiac output response to exercise results in an increase in anaerobic glycolysis with development of a lactic acidosis at low work rates. The lactic acidosis produces acid stimuli that increases ventilatory drive [44].

Mitani et al. [25] suggested that it is necessary – when performing a CPET in PAH patients – to observe not only VO2 or VCO2, but also Ve/VCO2, in order to prevent aggravation of the ventilation/perfusion inequality, which leads to exercise-induced hypoxemia. A higher VE/VCO2 ratio describes a greater ventilatory requirement for eliminating the CO2 produced by aerobic metabolism and defines a reduced ventilatory efficiency. The reduced ventilatory efficiency is therefore caused by an increase in physiological dead space and a reduced PaCO2 set-point [45]. Ting et al. [23] demonstrated that patients with higher Ve/VCO2 ratio would be patients with higher pulmonary vascular resistance. On the other side, the authors showed that the improvement in VE/VCO2 ratio paralleled the improvement in VD/VT following dosing with prostanoids. This supports the concept that a reduction in Ve/VCO2 ratio reflects an improvement in blood flow to the ventilated lung. Reybrouck et al. [24] found that the Ve/VCO2 slope was steeper in patients with PH than the slope in patients with normal PAP, and these authors found a significant correlation between the slope and PAP and the VD/VT. Because of ventilation-perfusion inequalities, the VD/VT is increased, making gas exchange less efficient than normal. This causes PetCO2 to be diluted relative to PaCO2. Because pulmonary vascular disease is the hallmark of patients with PH, it is likely that the remarkably low PetCO2 seen in our patients with PH is partially due to underperfusion of ventilated lung.

Our data indicate that abnormally reduced ventilatory efficiency parameters are powerful and independent predictors of mortality in patients with PAH/CTEPH. Our study demonstrates that an increased ventilatory response to exercise is associated with a lower survival in patients with stable PAH. For several years, peak VO2 was the “gold standard” and remains widely used to risk stratify this type of patients [2]. However, the limitations of peak VO2 have prompted the search for other indices of CPET that can serve as alternative prognostic factors. The main disadvantage of peak VO2 is the need for maximal exercise, which may be difficult to achieve, particularly in PAH patients, whose daily activity levels are far below the effort required by the test. In addition, peak VO2 may be underestimated because of low patient motivation or because of premature termination of the test by the physician. Thus supplementary indices are needed to sharpen the risk stratification [46]. Unlike peak VO2, for example the Ve/VCO2 slope is generally independent from subject effort. In addition, variation in the Ve/VCO2 slope appears to rely closely on central function in patients with PH. The closer reliance the Ve/VCO2 slope has on cardiac performance may contribute to its prognostic accuracy relative to peak VO2.

## Conclusion

The ventilatory efficiency parameters are easily measurable and highly reproducible parameters obtained from CPET, and the prognostic value of a reduced ventilatory efficiency suggests higher than the peak VO2 alone. Thus, a rational and pragmatic risk stratification process should include both peak VO2 and ventilatory efficiency parameters, and in particular, the measurement of the Ve/VCO2 slope and ventilatory equivalent for carbon dioxide should be performed in patients with PAH/CTEPH. In summary, based upon these preliminary results, we propose that these indices of CPET might be helpful in the care of these patients and could play a role as a prognostic effort-independent marker in the evaluation of PAH/CTEPH patients.

## Abbreviations

AaDO2, Alveolar-arterial oxygen difference; AT, Anaerobic threshold; AUC, Area under curve; BMI, Body mass index; CI, Cardiac index; CPET, Cardiopulmonary exercise testing; CTEPH, Chronic thromboembolic pulmonary hypertension; FVC, Forced vital capacity; O2 pulse, Oxygen pulse; PAH, Pulmonary arterial hypertension; PAP, Pulmonary arterial pressure; PCWP, Pressure in wedge position; PH, Pulmonary hypertension; PVR, Pulmonary vascular resistance; RA, Right atrium pressure; RHC, Right heart catheterization; SO2, Oxygen saturation; TLC, Total lung capacity; TLco, Diffusing capacity for carbon monoxide; VCO2, Carbon dioxide output; Ve, Minute ventilation; Ve/VCO2, Ventilatory equivalent for carbon dioxide; Ve/VO2, Ventilatory equivalent for oxygen; Ve/VCO2 slope, Slope between minute ventilation and carbon dioxide production; VO2, Oxygen uptake.

## Competing interests

The authors declare that they have no competing interests.

## Authors’ contributions

Drs. SVS and B conceived and designed the study. Dr. S performed the statistical analyses and drafted the article. Drs. SF and B acquired the study data. All authors participated in interpreting the data and revising the manuscript for important intellectual content. All authors approved the final version of the manuscript.

## Pre-publication history

The pre-publication history for this paper can be accessed here:

http://www.biomedcentral.com/1471-2466/12/23/prepub
